# Response of phytoplankton to banana cultivation: A case study of Lancang-Mekong River, southwestern China

**DOI:** 10.1038/s41598-019-45695-x

**Published:** 2019-06-24

**Authors:** Juan Dai, Yinjun Zhou, Haipeng Wu, Yunchao Zhang, Kongxian Zhu

**Affiliations:** 1 Changjiang River Scientific Research Institute, Wuhan, 430010 P.R. China; 2 Key Laboratory of River Regulation and Flood Control of MWR, Wuhan, 430010 PR China; 3 College of Life Sciences, Hebei University, Baoding, 071002 P.R. China

**Keywords:** Biodiversity, Riparian ecology

## Abstract

This study examined the possible effects of banana cultivation on phytoplankton biomass and community structure in southwest China along the Lancang-Mekong River. Water and phytoplankton samples were collected on March (dry season) and August (rainy season), and physical-chemical properties of water, phytoplankton biomass and community structure were determined. The results indicated that the banana cultivation resulted in increases in sediment, total phosphorus (TP) and total nitrogen (TN) concentrations at estuaries of Lancang-Mekong River branches. Cultivation decreased phytoplankton diversity, abundance and biomass, as well as changed the phytoplankton community structure at estuaries of branches. Sediment concentration (increased by cultivation) was considered as the dominant influence factor of phytoplankton biomass and community structure. However, at downstream sites (primary channel), banana cultivation did not cause (result from its huge flow) the significant changes in physical-chemical properties of water, phytoplankton biomass or community structure.

## Introduction

Wetlands are one of the most important ecosystems of the world^1–3^, and play a critical role in climate change and biodiversity protection^4,5^. Phytoplankton are a key primary producer in freshwater wetlands and oceans^6–9^, and are crucial in global ecosystem structure and function^10–12^. They could also affect the population and diversity of other organisms throughout the food chain in that they are the initial primary producer of the food chain^13–15^. Some phytoplankton species, which incidentally are prominent noxious bloomers (such as: high pollution tolerant diatoms, dinoflagellates, and toxin producing cyanobacteria), could cause a deterioration of water quality and mortality of some fish species^15–17^. Phytoplankton are sensitive to changes in multiple environmental factors^8,18,19^. The interactions among turbulent mixing, underwater light availability, nutrient inputs, and grazing pressure can strongly effect the composition and diversity of phytoplankton, leading to strong and predictable succession patterns of phytoplankton in water bodies^8,20–23^. All these factors make phytoplankton suitable indicators of monitoring ecological transformations and their magnitude^14,24–26^.

Banana cultivation is one of the world’s main agriculture activities and is considered to be one that has the greatest impact on terrestrial ecosystems^27,28^. This cultivation is different from other agriculture activities in that the plants’ foliage shades the ground inhibiting the establishment of erosion resistant ground cover^29,30^, resulting in lack of water retention and subsequently soil erosion, with the runoff carrying chemical fertilizer and pesticide to water bodies^29^, affecting rivers and lakes ecosystems. Lancang-Mekong River basin is one of the key banana growing areas in the world, and Lancang-Mekong River is one of the largest rivers (located in the southeastern region of the Eurasian continent) in the world^31^.

Effects of banana cultivation on ecosystem and biodiversity are a research priority worldwide^27,32^. Such as, Corbi *et al*.^29^ studied the effect of banana cultivation on aquatic insect species, and found (1) number of organisms from streams in areas of banana cultivation was higher than that of control area (1105 and 706 individuals, respectively) from streams in preserved areas; and (2) the forested streams had higher richness and diversity of EPTC (Ephemeroptera, Plecoptera, Trichoptera and Coleoptera) than banana plantation streams. However, the little information that is available concerning the effects of banana cultivation on phytoplankton contained little biomass and diversity information.

In this study, we examined the responses of physical-chemical properties of water, biomass and diversity of phytoplankton to banana cultivation in Lancang-Mekong River basin. Based on the work, the objectives of the study were: (1) to analyze the effects of banana cultivation on biomass and diversity of phytoplankton; and (2) to increase our knowledge about the relationship among the water’s physicochemical properties, the biomass and diversity of phytoplankton, and anthropogenic activities (banana cultivation).

## Materials and Methods

### Study area

**Figure 1:**
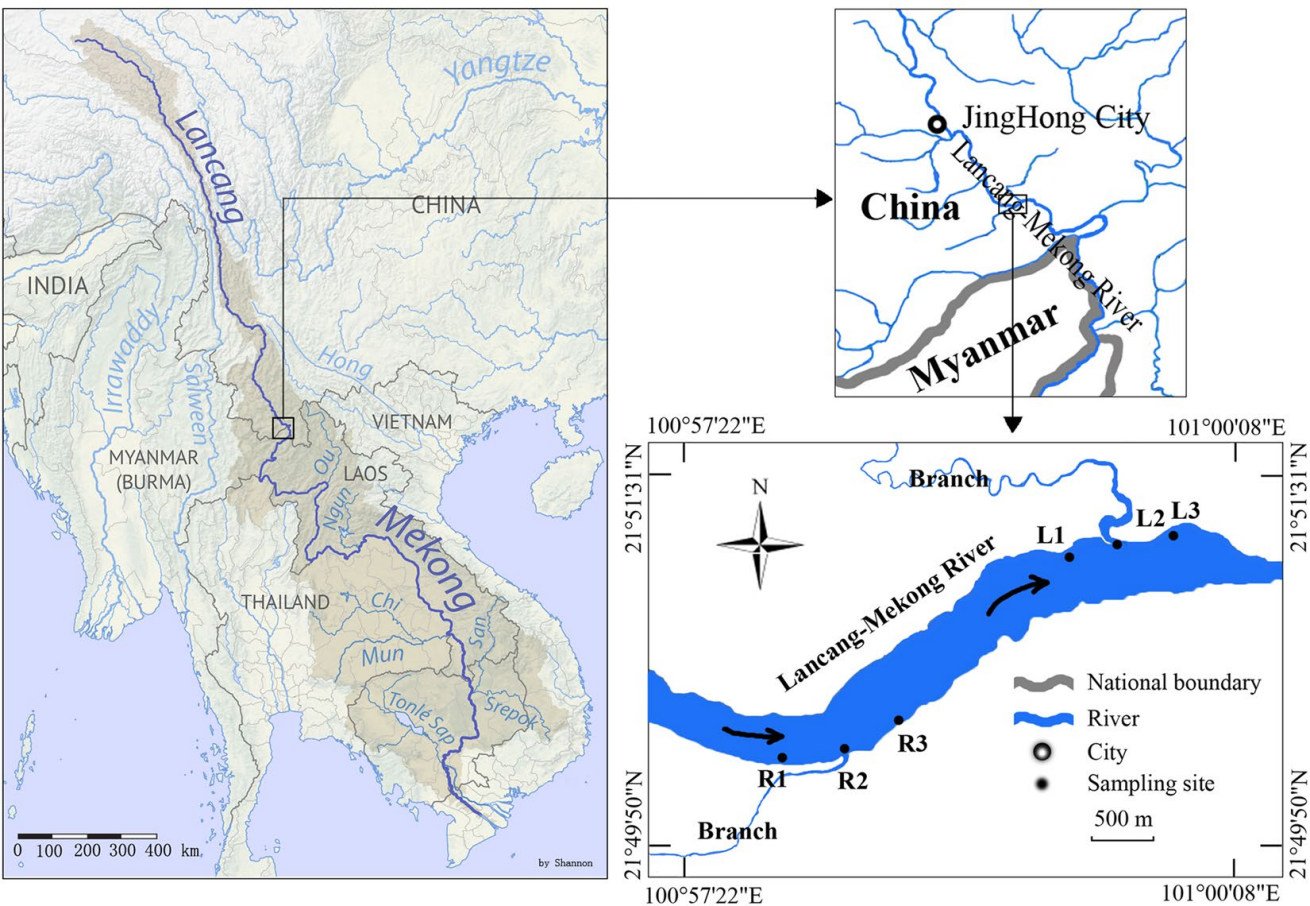
The map of sample sites in this study. The map of the Mekong river basin (Left part of Fig. 1) was finished by Shannon1 (https://commons.wikimedia.org/w/index.php?curid=65845951). We removed the cities from the origin map and increased font sizes of scale. This image is under the CC BY-SA 4.0 license (https://creativecommons.org/licenses/by-sa/4.0/).

Lancang-Mekong River is one of the largest rivers in the world^31,33^. It runs through southwestern China, Myanmar, Thailand, Laos, Cambodia and Vietnam^31,34^. Ganlanba is a basin valley, which is located at the middle regions of Lancang-Mekong River and its total area is about 56 km2. Banana cultivation is the main agriculture activity of this region. The banana seedlings are planted in spring or autumn. After 10~15 months, banana fruit is ripe for the first time and then will ripe each year for the next 3~5 years. The fields are frequently flooded (especially in the dry season, water is delivered to the field through the collapsible flexible plastic tube and dewatering of fields water directly flow into branch waterway) and many times’ fertilization and applying pesticide each year in the banana field. The climatic regime is composed of two seasons: the rainy season (from May to October) dominated by the southwest monsoon and the dry season (from November to following April) dominated by the mainland west monsoon^31,35^. There is one branch on each side of the river (Fig. 1). The agricultural effluent of this region flows into Lancang-Mekong River through the two branches.

### Sampling strategy

There were six sampling sites (R1, R2, R3, L1, L2 and L3) in this study (Fig. 1). “R” and “L” refers to right and left riverbank respectively, facing downstream. Sites R2 and L2 are estuary confluences of the two branches with the Lancang-Mekong River, where regional agricultural effluent flows into Lancang-Mekong River. Sites R1 and L1 are located approx. 500 m upstream of the confluence and are the control sites. Sites R3 and L3 are located approx. 500 m downstream of the confluence. The samples of these two sites represented the downstream samples affected by banana cultivation. Samples were collected from the three sampling sites of right riverbank in March 2015 (dry season) and the six sampling sites in August 2015 (rainy season). The first letter of the samples name indicated the sample was collected on March (M) or August (A), such as MR1 and AR1. Individual water samples were collected with water depth at 0.5, 1.0 and 2.0 m at each site, using a 3 L water sampler, then stored in washed polyethylene bottles. Water samples from each of the three different depths were pooled into single composite samples for subsequent physical-chemical properties analysis. Additionally, 12 L of water from each of the three depths were collected and poured into a phytoplankton net (35 μm-mesh size). The collected phytoplankton samples were immediately preserved with 1% Lugol’s solution in a labeled bottle on sites. Water samples and phytoplankton samples were carried into lab and sample analysis was initiated in 48 h.

### Analytical procedures for physical-chemical properties of water

The vertical profiles of the physical-chemical properties of each sample site, including the pH, temperature, dissolved oxygen (DO) and total dissolved solids (TDS, total content of inorganic salts and organic compounds dissolved in water, or the residue left after evaporating (105~110 °C) the filtered water to dryness), were measured *in situ* to determine the values of the mixed layer using the 6600V2 multiparameter meter (Yellow Springs Instruments, Ohio, USA). The total phosphorus (TP) was examined by ammonium molybdate tetrahydrate spectrophotometry^36^. The total nitrogen (TN) was determined by ultraviolet spectrophotometry^36^. The permanganate index (COD_Mn_) was measured according to acidity method^36^. Sediment concentration was measured by oven drying method.

### Phytoplankton identification and enumeration

Phytoplankton identification and enumeration was accomplished using a microscope (Olympus BX 51, Japan) at ×100 magnification according to Utermoeh method^37^. More than 100 fields of view were analyzed for each replicate, and the maximum counting error between two replicates was less than 20% upon the calculation of the standing stock of phytoplankton^8^. Cell volume of each species was estimated based on the average cell dimensions according to an appropriate volume formula^38^. The phytoplankton fresh biomass (μg L^−1^) was calculated according to cell volume (cm^3^ L^−1^) and water density (1.0 g cm^−3^)^8^. Calculation of biomass was performed at the species level.

### Statistical analyses

Phytoplankton community diversity was calculated by the Shannon-Weiner diversity index^2^. Correlation analysis was completed to determine the relationships between phytoplankton parameters and water parameters. It was performed using SPSS (version 11.5).

Canoco (version 4.5, Centre for Biometry, Netherlands) was used to examine the multivariate relationships between the phytoplankton community structure and the physical-chemical properties of water. The relative abundance of a species was calculated according to its number and the sum of numbers of all 44 species in each sample. It was used for the subsequent analysis. Detrended correspondence analysis (DCA) was performed to determine whether the data for the phytoplankton community structure followed a unimodal or linear response model. The maximum length of the DCA ordination axis was 4.948, which clearly indicated a unimodal species response. Accordingly, canonical correspondence analysis (CCA) with default settings was completed to ordinate the phytoplankton community structure (The relative abundance) with the physical-chemical properties of water^39^. Ordination biplots with scaling of inter-species differences displayed phytoplankton community structure similarities, so that the distances between each centroid points for sample were easily understood^2^. In forward selections, the Monte Carlo permutation test (499 unrestricted permutations) was performed to determine the parameters that significantly affected the phytoplankton community structure^39^.

## Results

### Effects of banana cultivation on physical-chemical properties of water

**Figure 2:**
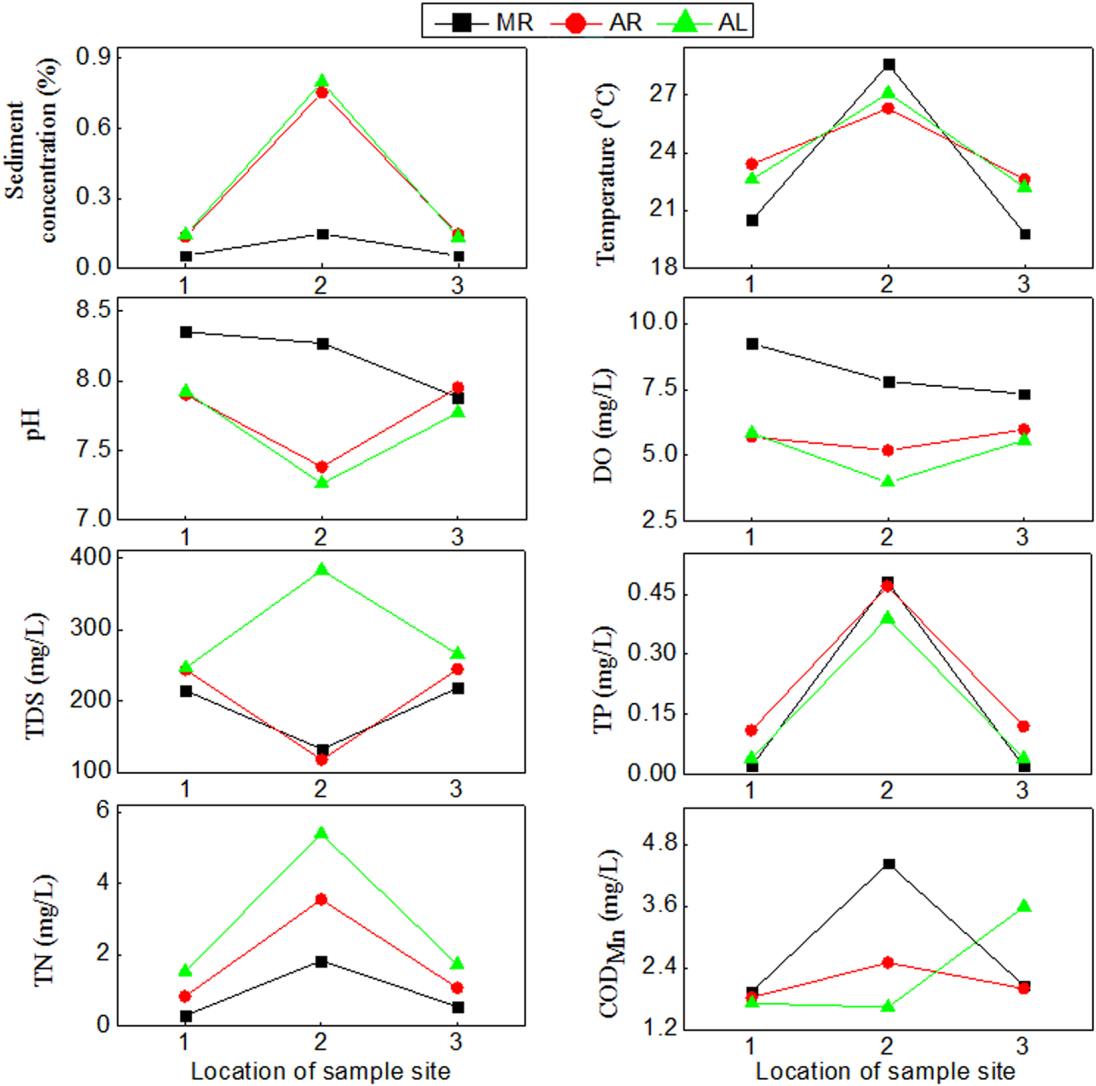
The physical-chemical properties of each water sample. DO: dissolved oxygen. TDS: total dissolved solids. TP: total phosphorus. TN: total nitrogen. CODMn: permanganate index. The value of abscissa showed the location of sample site: 1 - upstream of confluence; 2 - confluence of branch; 3 - downstream of confluence. MR: sample from right sample site was collected on March. AR: sample from right sample site was collected on August. AL: sample from left sample site was collected on August.

The physical-chemical properties of each water sample are shown in Fig. 2. The sediment concentration of all samples at estuaries of the branches (L2 and R2) was 1.7–5.0 times higher than those of all adjacent samples (L1 and L3, R1 and R3, respectively) at the same sampling time. The sediment concentration of sample on August was 1.5–4.1 times higher than that of the same sampling site on March (such as, MR1 and AR1, MR2 and AR2). Similar TP, TN and temperature changes occurred among these samples, as did the sediment concentration. The TP, TN and temperature of all samples at estuaries of the branches was (2.9–23 times, 2.1–5.3 times and 0.12–0.44 times, respectively) higher than those of all adjacent samples at the same sampling time. The sediment concentration, TP and TN of sample on August were usually higher than those of March of the same sampling site. However, the pH, DO, TDS and COD_Mn_ between samples at estuaries of the branches and adjacent samples had no significant differences. Almost all the physical-chemical properties of water at upstream and downstream sites were approximations.

### Effects of banana cultivation on phytoplankton abundance and fresh biomass

**Figure 3:**
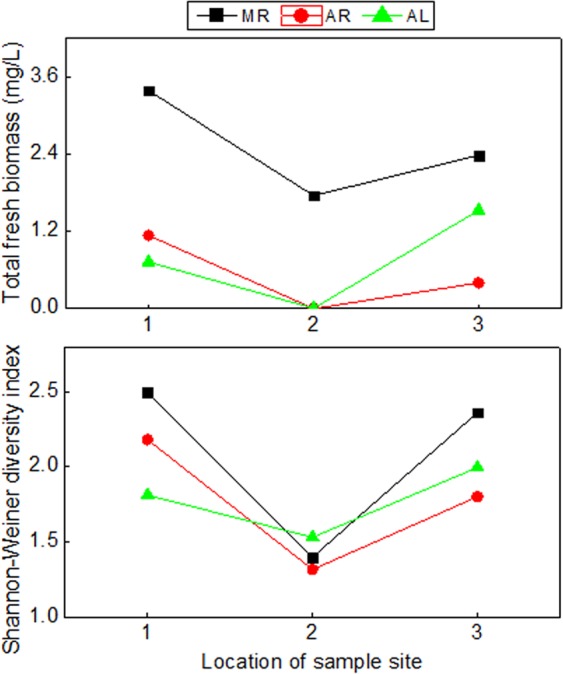
The phytoplankton total fresh biomass and Shannon-Weiner diversity index of each sample. The value of abscissa showed the location of sample site: 1 - upstream of confluence; 2 - confluence of branch; 3 - downstream of confluence. MR: sample from right sample site was collected on March. AR: sample from right sample site was collected on August. AL: sample from left sample site was collected on August.

**Figure 4:**
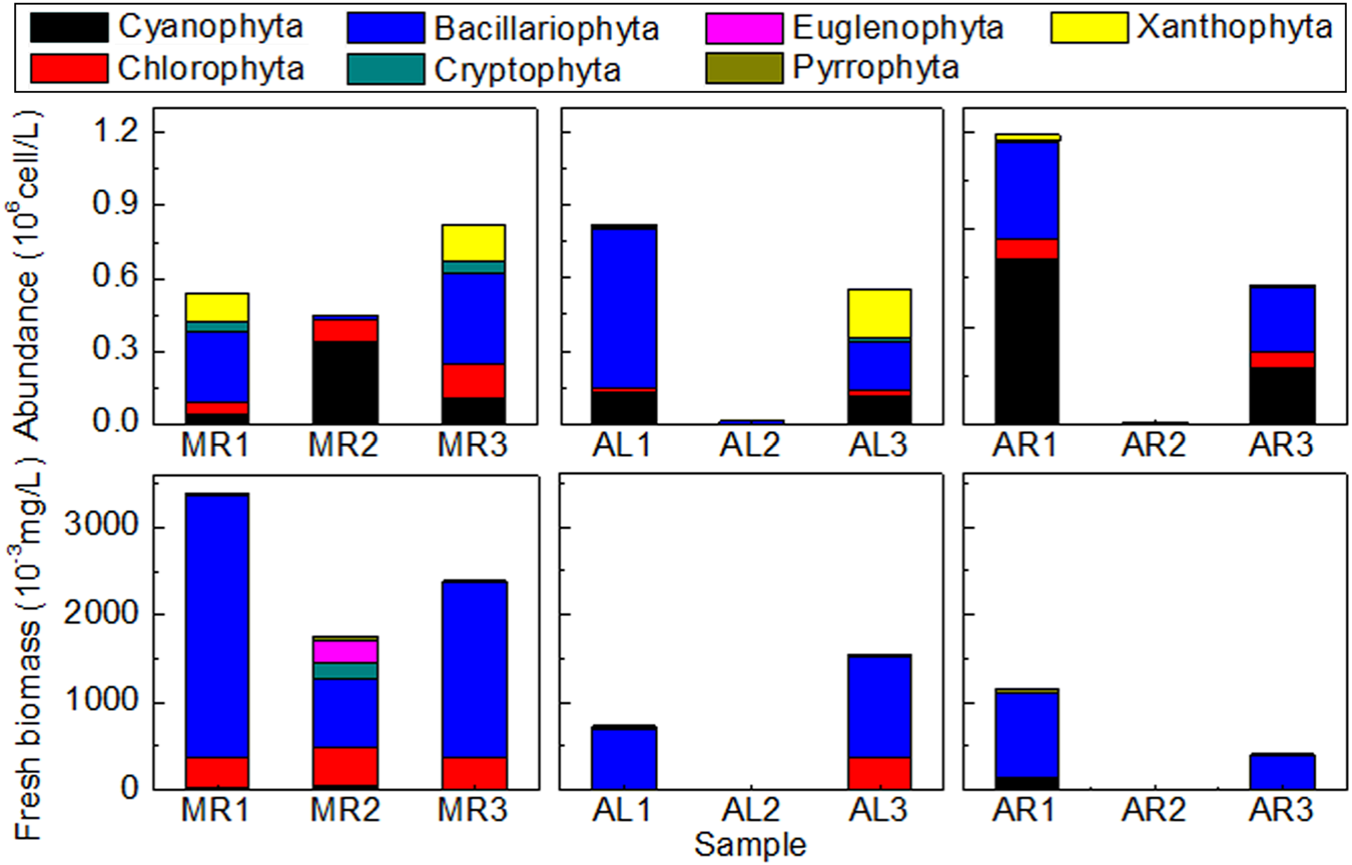
Abundance (10^6^ cell/L) and fresh biomass (10^−3^ mg/L) of each phylum of each sample. The value of abscissa showed the location of sample site: 1 - upstream of confluence; 2 - confluence of branch; 3 - downstream of confluence. MR: sample from right sample site was collected on March. AR: sample from right sample site was collected on August. AL: sample from left sample site was collected on August.

The total fresh biomass of phytoplankton of each sample is shown in Fig. 3. The total fresh biomass of phytoplankton of all samples at estuaries of the branches was lower (decreased by 26.4–99.9%) than that of all adjacent samples at one same sampling time. The total fresh biomass of phytoplankton of sample on August was lower (decreased by 66.38–99.95%) than that of the same sampling site on March. The total fresh biomass of phytoplankton of samples between upstream site and downstream site were approximate. The abundance of the phytoplankton of each sample had the similar changes. The abundance and fresh biomass of *Cyanophyta*, *Chlorophyta*, *Bacillariophyta*, *Cryptophyta*, *Euglenophyta*, *Pyrrophyta* and *Xanthophyta* of each sample is shown in Fig. 4. *Cryptophyta* had the maximum fresh biomass in AR2, *Bacillariophyta* had the maximum fresh biomass in other samples.

The correlations between the total fresh biomass of phytoplankton and physical-chemical properties of water are shown on Table 1. The total fresh biomass of phytoplankton was strongly negatively correlated with the sediment concentration and TN. However, there was a significant positive correlation between the total fresh biomass of phytoplankton and the pH or DO. Besides, the temperature, TDS, TP and COD_Mn_ were not correlated with the total fresh biomass of phytoplankton.

**Table 1:** Pearson’s correlation coefficients between the parameters.

	Total biomass	Shannon-Weiner Diversity Index
Sediment concentration	−0.705^*^	−0.685^*^
Temperature	−0.554 ^*ns*^	−0.890^***^
pH	0.767^*^	0.490 ^*ns*^
DO	0.901^***^	0.537 ^*ns*^
TDS	−0.226 ^*ns*^	0.181 ^*ns*^
TP	−0.500 ^*ns*^	−0.871^**^
TN	−0.705^*^	−0.735^*^
CODMn	0.180 ^*ns*^	−0.361 ^*ns*^

Significance levels are indicated by *(*p* < 0.05), **(*p* < 0.01), ***(*p* < 0.001), while *ns* indicates no significant correlation (*p* > 0.05).

### Effects of banana cultivation on phytoplankton community structure

The Shannon-Weiner diversity index of phytoplankton of each sample is shown in Fig. 3. The Shannon-Weiner diversity indexes of all samples at estuaries of the branches were lower than that of all adjacent samples at one same sampling time, and decreased by 15.6–44.1%. The Shannon-Weiner diversity indexes of sample on August were lower than that of the same sampling site on March, and decreased by 5.9–23.7%. The correlations between Shannon-Weiner diversity index and physical-chemical properties are shown on Table 1. There was a significant negative correlation between the Shannon-Weiner diversity index and the sediment concentration, temperature, TP or TN. However, the pH, DO, TDS and COD_Mn_ were not correlated with Shannon-Weiner diversity index.

**Figure 5:**
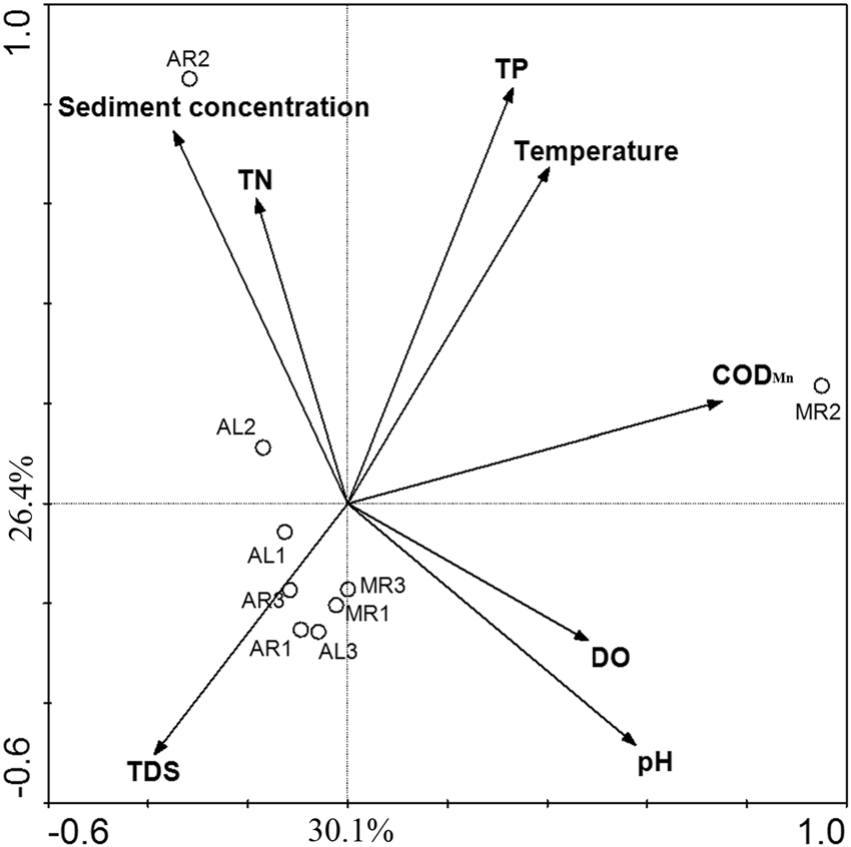
Canonical correspondence analysis for phytoplankton community structure and physical-chemical properties of water. Samples are indicated by open circles. Physical-chemical properties of water are represented by solid lines with filled arrows.

The CCA biplot of the phytoplankton community structure and the investigated physical-chemical properties of water are shown in Fig. 5. As shown in Fig. 5, the phytoplankton community structure of the samples at estuaries of the branches (MR2, AL2 and AR2) had significant differences from those of all adjacent samples. The phytoplankton community structure of the samples of upstream of confluence was similar to that of downstream of confluence. Table 2 is showing that all of the physical-chemical properties could explain 82.9% of the variation in the community-environment relationship. The TP, temperature and sediment concentration exerted highly significant influences on the phytoplankton community structure. Each physical-chemical property of water explained a different aspect of the variation in the community-environment relationship. The community-environment relationship variation explained in by the physical-chemical properties of water decreased as follows: TP > temperature > sediment concentration >COD > pH > TDS > TN > DO. All of the aforementioned results of correlation analysis and CCA suggested that the TP, temperature, sediment concentration were the dominant influence factors of the phytoplankton community structure.

**Table 2:** The results of Monte Carlo permutation test for the test of influence of the physical-chemical properties.

Parameters	% Variation explains	P-value
TP	23.3	0.002
Temperature	20.9	0.012
Sediment concentration	20.9	0.048
COD_Mn_	20.8	0.106
pH	19.0	0.108
TDS	17.0	0.202
TN	15.9	0.226
DO	15.3	0.282
All above together	82.9	

## Discussion

### Effects of banana cultivation on physical-chemical properties of water

Our results indicated that the banana cultivation caused the increases of the sediment concentration, TP and TN at estuaries of the branches of Lancang-Mekong River. This is because of the bare ground of banana fields, which caused by maintenance of the vegetal covering^29,40^. And rainfall and irrigation on this bare ground caused seriously water and soil erosion, which carried lot of sediment, phosphorus and nitrogen into rivers. However, because flow of the trunk stream (about 1500~3000 m^3^/s) was much larger than that of branches (about 30~70 m^3^/s), the banana cultivation could not cause the significant changes of sediment concentration, TP and TN at downstream sites. We also found the sediment concentration, TP and TN of sample on August were usually higher than those of the same sampling site on March. It was because that more frequent and intense rainfall in the rainy season caused more seriously water and soil erosion. Besides, the temperature at estuaries of the branches was higher than those of all adjacent samples at one same sampling time maybe because the trunk stream had a greater depth than that of the branches which caused lower temperature at the bottom.

### Effects of banana cultivation on phytoplankton

The results of this study showed that the banana cultivation caused the decreases of the total fresh biomass and diversity of phytoplankton at estuaries of the branches. The correlation analysis and CCA suggested that the TP, temperature, sediment concentration were the dominant influence factors of the phytoplankton community structure. And sediment concentration was significantly negatively correlated with the total fresh biomass and the Shannon-Weiner diversity index of phytoplankton. The TP and temperature were significantly negatively correlated with the Shannon-Weiner diversity index of phytoplankton.

Other studies also supported that the total fresh biomass and diversity of phytoplankton were limited by higher sediment concentration^7,15,41^, which was caused by banana cultivation in this study. This was because that higher sediment concentration caused lower transparency, which could limit the light penetration. High light could lead to more photosynthesis and increase of phytoplankton biomass^42–44^. But phytoplankton in the turbid water suffered from severe light limitation^7^. Other studies also found that the dominant species competitiveness with light limiting corresponded to lower diversity^15,45,46^. Chen *et al*.^47^ also reported that the decrease of turbidity and enhancing of light penetration could promote the growth of phytoplankton.

In this study, the TP and temperature were significantly negatively correlated with diversity of phytoplankton. It was inconsistent with the following viewpoints: (1) higher nutrient (nitrogen and phosphorus) and resource availability can stimulated higher activity of phytoplankton, and resulted in higher abundance and diversity of phytoplankton^7,15,42,48^; and (2) higher temperature promoted growth of phytoplankton, which would result in higher abundance and diversity^7,49^. This different was a result of that the effects of phosphorus and temperature on phytoplankton dynamics could modulate by other factors (such as light and sediment concentration)^42^. Other study also found that higher sediment concentration could limit the light penetration and phytoplankton production despite the high nutrient content^15,50^. Therefore, the sediment concentration may be the dominant influence factor of phytoplankton biomass and community structure in this study. The banana cultivation caused the increases of the sediment concentration at estuaries of the branches and no obvious changes of that at downstream sites, and then caused the changes of phytoplankton further.

Considering the crucial function of phytoplankton to global ecosystem, changes of phytoplankton at estuaries of the branches (which was caused by banana cultivation) mean a series of ecological changes in the estuaries and channels of the branches. And further study is required to examine these changes. Besides, no significant change was found in the phytoplankton at downstream sites. This is because that the flow of the trunk stream of Lancang-Mekong River (about 1500~3000 m^3^/s) is much higher than those of the branches (about 30~70 m^3^/s), and the huge flow of the trunk stream diluted the changes of water quality and phytoplankton (caused by banana cultivation). However, the larger scale of banana cultivation may cause some changes of phytoplankton at downstream sites of the trunk stream of Lancang-Mekong River, which also need further study. Besides, the different responses of phytoplankton in euphotic zone and below euphotic zone to banana cultivation also need further study.

## Conclusions

The following conclusions were drawn on this paper: the banana cultivation caused the increases of the sediment concentration, TP and TN at the estuaries of branches. And these increases caused the decrease of the phytoplankton diversity, abundance and fresh biomass, and the changes of phytoplankton community structure. Sediment concentration was considered as the dominant influence factor of phytoplankton biomass and community structure. Banana cultivation did not result in material differences in physical-chemical properties of water, phytoplankton biomass or community structure at downstream sites due to disproportional volumetric flows and resultant dilution of the tributary component.
